# The normal function of the cancer kinase Mirk/dyrk1B is to reduce reactive oxygen species

**DOI:** 10.18632/genesandcancer.1

**Published:** 2014-01

**Authors:** Xiaobing Deng, Stephen E. Mercer, Chi-Yu Sun, Eileen Friedman

**Affiliations:** ^1^ Pathology Department, Upstate Medical University, Syracuse, New York

**Keywords:** Mirk/dyrk1B, ROS, fast twitch muscle, antioxidant genes

## Abstract

Mirk kinase is a gene upregulated and sometimes amplified in pancreatic cancers and in ovarian cancers, but expressed at very low levels in most normal diploid cells except for skeletal muscle. The muscle cell function of Mirk kinase selected for by cancer cells is unknown. It is now shown that Mirk protein is expressed at low levels and is largely nuclear in cycling skeletal muscle C2C12 myoblasts, but is translocated to the cytoplasm and upregulated when myoblasts initiate differentiation, as shown by immunofluorescence staining and by cell fractionation. Either Mirk depletion or Mirk kinase inhibition increased ROS levels in cycling C2C12 myoblasts. However, Mirk protein is localized in the cytoplasm of mature muscle fibers, specifically in the fast twitch fibers of human skeletal muscle where toxic ROS levels are generated by muscle contraction. C2C12 myoblasts at high density in differentiation media fuse to form differentiated postmitotic myotubes that can contract. A Mirk kinase inhibitor induced a dose-dependent increase in ROS in this model for fast twitch fibers of human skeletal muscle. Efficient Mirk depletion in SU86.86 pancreatic cancer cells by an inducible shRNA decreased expression of eight antioxidant genes. Thus both cancer cells and differentiated myotubes utilize Mirk kinase to relieve oxidative stress.

## INTRODUCTION

Mirk/dyrk1B is a gene upregulated and sometimes amplified in the majority of pancreatic cancers and in ovarian cancers [1]. However, Mirk is a skeletal muscle kinase, so it was unclear what muscle function is maintained in these cancer cells. Mirk is not an essential gene because embryonic knockout of Mirk/dyrk1B caused no evident phenotype in mice [2]. Skeletal muscle development appeared normal, so it is possible that other members of the dyrk family of related serine/threonine kinases were upregulated in the Mirk embryonic knockout mice. Mirk has greatest abundance and activity in normal diploid cells and in cancer cells transiently arrested in G0, or in early G1, with up to 10-fold lower levels in cycling cells [3]. Mirk is sometimes considered a G0-kinase because, in poor growth conditions, Mirk mediates the arrest of cells in G0 by destabilizing the cyclin D family, preventing exit into G1, and by stabilizing the CDK inhibitor p27 needed for G0 arrest [4],[3]. However, Mirk G0 function may not be significant in skeletal muscle development. Several investigators have shown that exit of undifferentiated myoblasts into G0 prevents differentiation, including myogenin induction [5], and the increase in the myogenin transcription factors and chromatin binding factors, including MyoD, p68, p300 and p8, occurs in mid-G1 [6], [7].

Mirk/dyrk1B is a multifunctional serine/threonine kinase that plays critical roles in muscle differentiation by regulatory effects on cell cycle progression, transcription, and cell survival [8], [9]. The Mirk protein contains a bipartite nuclear localization sequence and during myogenesis, Mirk targets effector molecules in the nucleus to promote differentiation [8], [10]. Mirk promotes myoblast differentiation indirectly by phosphorylating class II histone deacetylases, causing them to accumulate in the cytoplasm and thus relieving suppression of myogenin-dependent transcription [10]. Mirk acts to promote the survival of differentiating myoblasts, at least in part, by phosphorylating the CDK inhibitor p21, causing p21 to accumulate in the cytoplasm where it functions as an anti-apoptotic signaling molecule [11]. However, p21 is often at low abundance in cancer cells, in particular when p53 is mutated or inactivated, so some other role of Mirk in normal muscle cell survival was sought.

## RESULTS

### The kinase Mirk/dyrk1B is found predominantly in the cytoplasm of differentiating C2C12 myoblasts and in adult skeletal muscle

**Fig 1:**
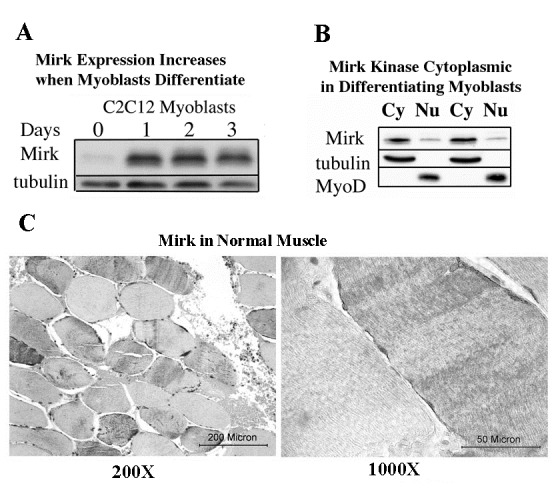
Mirk localizes in the cytoplasm of differentiating C2C12 myoblasts and Mirk restriction to the cytoplasm persists in adult human skeletal muscle. (A) Mirk expression increases when myoblasts differentiate. Mirk was detected by western blotting in C2C12 myoblasts in growth medium (day 0) or in differentiation medium for 3 days (days 1-3).(B) Mirk is cytoplasmic in differentiating myoblasts. Duplicate cultures of C2C12 myoblasts were cultured in differentiation medium for 1 day before partition into nuclear and cytoplasmic fractions and analysis of Mirk protein localization by western blotting. Fractionation was monitored by Western blotting for tubulin as the cytoplasmic (Cy) marker and MyoD as the nuclear (Nu) marker. (C) Paraffin-embedded sections of adult human muscle were analyzed by immunohistochemistry with anti-Mirk C-terminal antibody. Note that Mirk is found in only a subset of fibers, where it is localized exclusively in the cytoplasm and often enriched in the perinuclear region.

**Fig 2:**
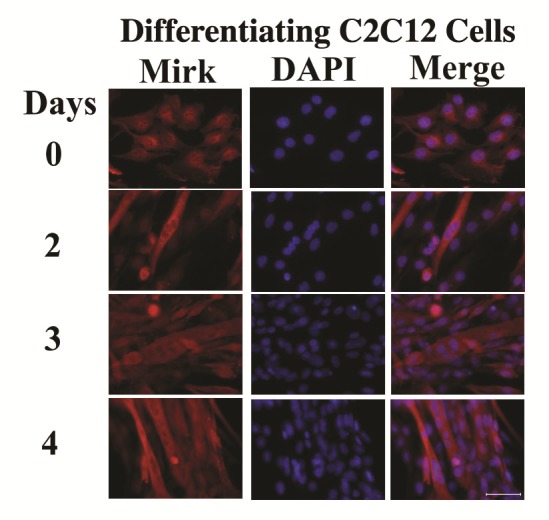
Mirk becomes restricted to the cytoplasm during myogenesis Localization of endogenous Mirk in cultured C2C12 mouse myoblasts by immunofluorescence analysis using affinity-purified antibody to the Mirk C-terminus and anti-rabbit secondary antibody conjugated to Alexa Fluor 594. Endogenous Mirk was detected in C2C12 myoblasts in growth medium (day 0) or in differentiation medium for 3 days (days 2-4). Nuclei were visualized with DAPI. Original magnification 400X. Scale bars = 50 μm.

The C2C12 in vitro model of myogenesis was used to characterize the functions of Mirk kinase in normal diploid cells[8], [9]. Mirk is expressed at very low levels in cycling cells and in most normal tissues except for skeletal muscle [12]. Mirk is expressed at higher levels in differentiating C2C12 myotubes where it mediates survival [10]. Mirk protein levels increased a mean of 13-fold when C2C12 myoblasts were placed in serum restricted differentiation medium (Fig.1A). Endogenous Mirk was expressed at a low level in cycling C2C12 mouse myoblasts (Fig.1A), and was located diffusely throughout the cell, but primarily in the nucleus and perinuclear region (Fig. 2, top panels). In sharp contrast to the pancellular distribution of Mirk in cycling myoblasts, a dramatic shift in the localization of Mirk to a solely cytoplasmic location was seen in differentiating G1-arrested myotubes within 2 days of shift to differentiation medium (Fig. 2, lower panels).

Subcellular fractionation of differentiating myoblasts confirmed that Mirk is primarily cytoplasmic in differentiating myoblasts, with an average of 6-fold more Mirk protein in the cytoplasmic fractions (Fig. 1B). Duplicate cultures of C2C12 myoblasts were cultured in differentiation medium for 1 day before partition into nuclear and cytoplasmic fractions and analysis for the presence of Mirk protein by western blotting. Later time points were not examined because the multinucleated myotubes proved very difficult to fractionate. The anti-C-terminal Mirk antibody used detects all of the splice variants of Mirk with known kinase activity [2] in a variety of cell lines under both growth and differentiation conditions (data not shown). Immune-complex kinase assays also had demonstrated that this antibody detects the active kinase in multiple cell types [13]. Traditional immunohistochemistry on formalin-fixed paraffin sections of adult human muscle was performed to determine the distribution of Mirk in mature myofibers. Mirk was restricted to the cytoplasm of adult muscle fibers (Fig. 1C). No staining was noted in control sections treated with non-specific rabbit IgG instead of primary antibody (data not shown). Identical patterns of localization were observed using our two distinct anti-Mirk antibodies in immunofluorescence analysis of frozen sections of adult human muscle (data not shown), thus demonstrating that the findings did not occur due to a non-specific binding of either antibody.

### Mirk/dyrk1B is found predominantly in the cytoplasm of fast-twitch fibers in adult skeletal muscle that produce reactive oxygen species during contraction

**Fig 3:**
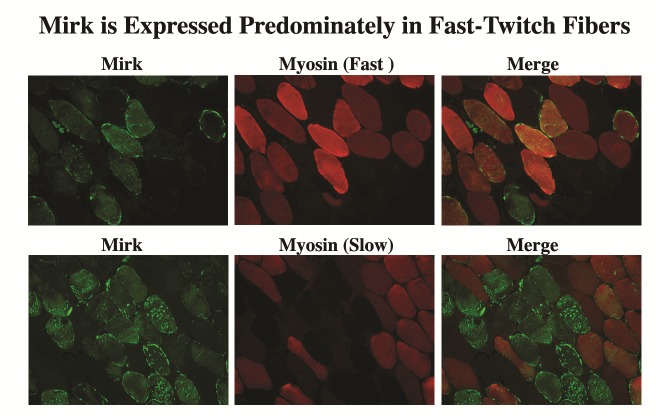
Mirk is expressed predominantly in fast-twitch fibers in adult human muscle Paraffin-embedded sections of adult human skeletal muscle were simultaneously incubated with rabbit polyclonal antibody against the unique Mirk C-terminus and a monoclonal mouse antibody against either the slow-twitch or the fast-twitch specific isoforms of myosin. Mirk labeling was subsequently visualized with an Alexa Fluor 488 conjugate (green), while the myosin isoforms were visualized with Alexa Fluor 594 (red). Original magnification 200X.

Fast twitch skeletal muscle endogenously produces ROS in response to repeated contractions [14]. Colocalization studies of Mirk with fast and slow myosin isoforms demonstrated that Mirk is expressed primarily in fast twitch fibers (Fig. 3). Identical patterns of localization were observed using immunofluorescence analysis of frozen sections of adult human muscle (data not shown). We have previously shown that Mirk plays a critical role in myoblast survival during differentiation [11]. The localization of Mirk in the cytoplasm of adult fast-twitch muscle fibers suggests that Mirk continues to play a role in adult muscle cell survival, perhaps by modulating the response to metabolic stress due to ROS produced during muscle contractions.

### Mirk kinase inhibitor acts like Mirk depletion

**Fig 4:**
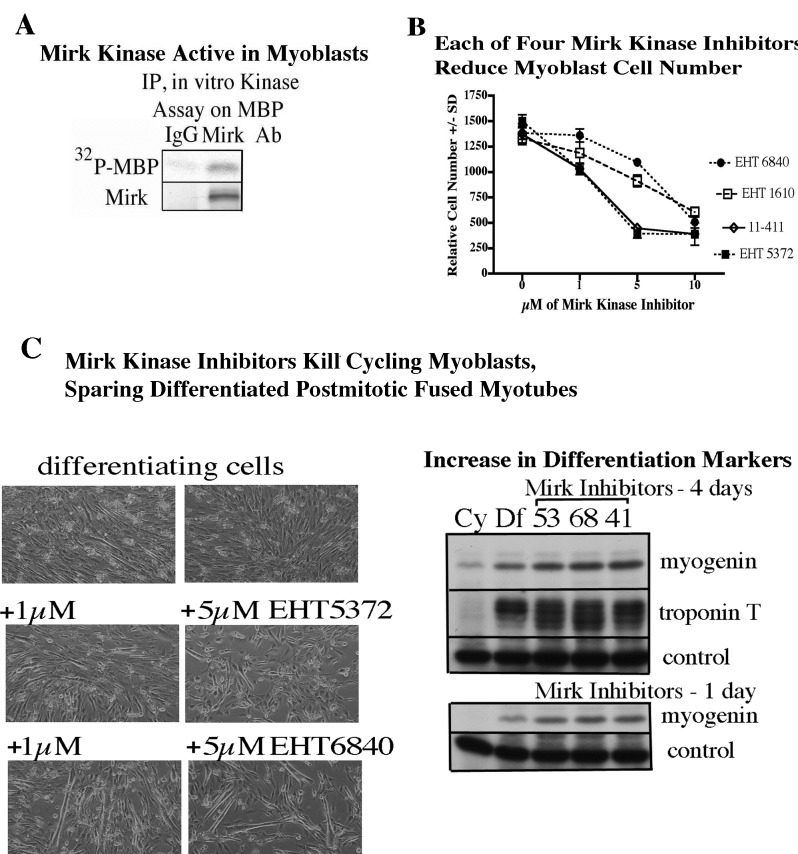
Since Mirk kinase mediates myoblast survival during differentiation, a Mirk kinase inhibitor should kill differentiating C2C12 myoblasts. (A) Mirk was immunoprecipitated from C2C12 myoblasts overnight with either an N'terminal directed affinity-purified polyclonal antibody to Mirk or with rabbit purified IgG as control. The kinase activity of immunoprecipitated Mirk, after collection on protein-A agarose beads was determined by an immune complex kinase reaction on myelin basic protein (MBP), which was analyzed by SDS-PAGE followed by autoradiography.(B) C2C12 myoblasts were cultured in differentiation medium for 3 days with increasing concentrations of the Mirk kinase inhibitors EHT5372, EHT6840, EHT1610, or 11-411. Mean+/−SD shown after measurement of cell number by MTT metabolism. (C) (left) C2C12 cells were placed in differentiation medium for 3 days and treated with Mirk inhibitors as shown. (right) Parallel cultures to panel B after 1 and 4 days of culture in differentiation medium were analyzed by western blotting for the transcription factor myogenin which controls myogenesis, the late maturation marker, fast twitch troponin T and a nonspecific band in the troponin blot as a western blotting control. Cy, cycling culture in growth medium; Df, culture in differentiation medium; Mirk inhibitors 53 (EHT 5372), 68 (EHT 6840), and 41 (11-411). Enrichment in differentiated, fused myoblasts should increase the level of differentiation markers.

Mirk is an active kinase in C2C12 cells, as shown by the ability of immunopreciptated Mirk to phosphorylate myelin basic protein (Fig.4A). Mirk maintains the survival of cycling myoblasts during the initial steps of differentiation as shown by Mirk depletion by RNAi [11]. Similarly to Mirk depletion, Mirk kinase inhibitors should reduce the viability of differentiating myoblasts. This was observed as each of four Mirk kinase inhibitors reduced the numbers of differentiating myoblasts (Fig 4B). Visual inspection revealed that single myoblasts were less in evidence in the cultures treated with the higher concentrations of Mirk kinase inhibitors, although large, fused differentiated post-mitotic myotubules remained in evidence (Fig.4C). Analysis of the muscle regulatory transcription factor myogenin, which mediates myoblast differentiation into myotubules, and the late maturation marker, fast twitch troponin T, showed that Mirk kinase inhibition with 3 different Mirk inhibitors during differentiation led to an increase in each differentiation marker compared to the differentiated myoblast culture (Fig.4C, right), consistent with the enrichment of differentiated myotubules because of loss of the undifferentiated cycling myoblasts. The increase in myogenin levels in the Mirk kinase inhibitor treated cultures was more marked after 1 day of differentiation, while the increase in the later induced troponin T levels was seen after 4 days. Thus the Mirk kinase inhibitors induced phenotype similar to Mirk depletion.

### Inhibition of Mirk kinase activity or Mirk depletion in C2C12 myoblasts leads to increased ROS levels

**Fig 5:**
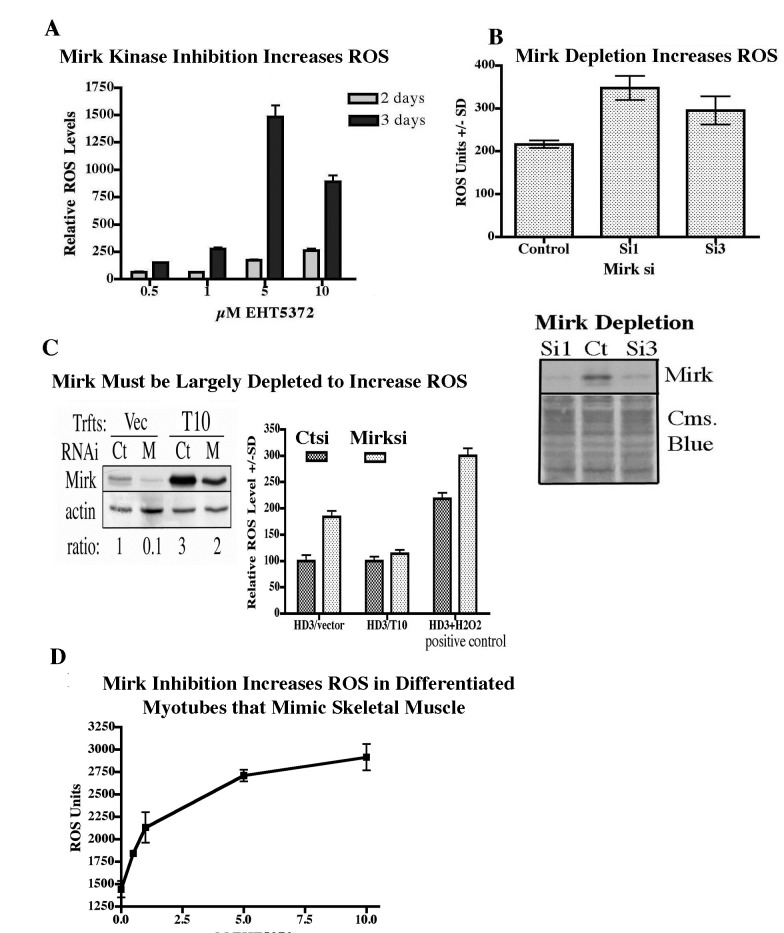
ROS levels increased after Mirk depletion or Mirk kinase inhibition in differentiating C2C12 myoblasts, a model for muscle fibers. (A) C2C12 myoblasts were cultured in differentiation medium for 2 or 3 days with increasing concentrations of the Mirk kinase inhibitor EHT5372. Four parallel cultures were assayed for each data point, with only two cultures receiving the CH-H2DCFA ROS indicator dye (5μM), and the others serving as controls. ROS values subtracted for control cultures not treated with Mirk kinase inhibitors. Mean of two separate experiments +/−SD. (B) C2C12 myoblasts were differentiation medium for 1 day, then transfected for 1 day with either pSilencer plasmid pSiM1 (directed to exon 3 of the murine Mirk/dyrkB gene) or pSiM3 (directed to exon 10) or pSiM2 as the negative control (directed to exon 3 but mutant at 2 positions). Four parallel cultures were assayed for each data point as in panel A. (lower panel) Parallel cultures analyzed for Mirk protein levels, with area of the blot then stained with Coomassie brilliant blue to show loading of the gel.(C) Mirk was depleted in the stable Mirk transfectant of the HD3 colon cancer lines, T10, and the vector control transfectant with synthetic RNAi duplexes siA (M lanes) or a GC-matched control, then examined for Mirk and actin levels by western blotting. (right panel) After Mirk depletion, ROS levels were measured as in panel A. One set of HD3/vector transfectant cells were treated with 100μM hydrogen peroxide for 4 hours as a positive control. Mean +/− SD shown, n=3.(D) C2C12 myoblasts were plated at high density and cultured until they were confluent and fused to form differentiated myotubes capable of contraction like skeletal muscle fibers. Cultures were then treated for 24 hr with increasing concentrations of the Mirk kinase inhibitor EHT 5372. Four parallel cultures were assayed for each data point as in panel A. Mean +/−SD shown.

Treatment with increasing concentrations of the Mirk kinase inhibitor EHT 5372 led to a dose-dependent increase in ROS values in differentiating myoblasts after 2 or 3 days (Fig.5A). Differentiating C2C12 myoblasts were transiently transfected with pSilencer plasmids expressing either Si1 to a sequence in murine Mirk cDNA within exon 3, Si3 to a sequence in exon 10, or a mutant sequence [3]. Depletion of Mirk by Si1 or Si3 led to an average 5.6 fold decrease in Mirk protein levels and to a 60% increase in ROS levels (Fig.5B). Thus both Mirk depletion and Mirk kinase inactivation led to an increase in ROS generation in differentiating myotubules, a model for skeletal muscle.

However, Mirk kinase inactivation was much more effective than Mirk depletion in inducing ROS. Possibly the Mirk kinase inhibitors affect other kinases which modify ROS levels. Alternatively, Mirk is a rather stable protein which dimerizes in vivo and is found in large molecular weight complexes over 660 kDa [15] where it maintains the integrity of the DREAM complex consisting of p130/Rb2, cyclin D, CDK4, CDK inhibitors, and other proteins [16]. In addition, Mirk phosphorylates class II histone deacetylases, causing them to accumulate in the cytoplasm and thus relieving suppression of myogenin-dependent transcription [10]. Mirk may half a long half-life in these complexes, while Mirk depletion may reduce the amount of any non-complexed Mirk molecules. Since Mirk is a kinase and is at high abundance in myoblasts, as much as in Panc1 cells where the Mirk gene is amplified, Mirk depletion may not remove enough Mirk to see an effect, while a small molecule inhibitor could diffuse throughout the cell to reach many Mirk molecules.

To test this hypothesis, the effect of knockdown of Mirk by synthetic duplex RNAi was tested in two stably transfected clones of the HD3 colon carcinoma cell line, the vector control transfectant and stable Mirk T10 transfectant expressing more Mirk protein [12]. After Mirk knockdown, the vector transfectant had only 10% of the original level of Mirk, while Mirk levels in the stable transfectant were only reduced 30% (Fig.5C). The 10-fold knockdown of Mirk in the vector control line led to a 2-fold increase in ROS levels (Fig.5C), similar to the increase in ROS levels seen after introduction of a K-ras mutation [17]. The 30% knockdown of Mirk in the Mirk stable transfectant line still left 20 times as much Mirk as in the vector control transfectant, and no increase in ROS was seen. As a positive control, the vector control line was treated with 100 μM hydrogen peroxide for 4 hours resulting in a 2-fold increase in ROS levels. These levels were increased slightly by Mirk knockdown. Thus colon cancer cells depleted of Mirk exhibited a 2-fold increase in ROS levels, similar to the increase seen when two pancreatic cancer cell lines were depleted of Mirk by either RNAi duplexes or an inducible shRNA to Mirk [18].

The experiments thus far have shown that either Mirk kinase inhibition or Mirk depletion reduce the viability of differentiating myoblasts by increasing the intracellular levels of toxic ROS. However, Mirk protein is localized in fast twitch fibers of postmitotic, polynuclear mature skeletal muscle. C2C12 myoblasts at high density in differentiation media will fuse to form differentiated postmitotic myotubes that can contract. A Mirk kinase inhibitor induced a dose-dependent increase in ROS in such postmitotic cultures (Fig.5D) without evident loss of cells. Thus Mirk acts to reduce ROS in this model of fast twitch skeletal muscle fibers.

### Depletion of Mirk by an inducible shRNA in SU86.86 pancreatic cancer cells shows loss of antioxidant combating activity

To identify antioxidant genes which were downregulated when Mirk levels were depleted, we utilized SU86.86 pancreatic cancer cells stably expressing a doxycycline-inducible shRNA to Mirk, in which ROS levels previously had been shown to increase when Mirk was depleted about 8 to 10-fold [18]. Eight genes with known antioxidant properties were downregulated when Mirk was depleted for 4 days in these cells, as measured by quantitative RT-PCR assaying a series of antioxidant genes (Table I). Hydrogen peroxide is a strong oxidant within cells. Three peroxidases are upregulated by Mirk, glutathione peroxidase (GPX2) a major hydrogen peroxide reducer in the GI tract, peroxidasin (PXDNL), and peroxiredoxin 2 (PRDX2). The latter reduces hydrogen peroxide and alkyl hydroperoxides while another Mirk-regulated gene sulfiredoxin (SRXN1) reactivates hyperoxidized peroxiredoxin through reduction of cysteine sulfinic acid in the active site. Lipoxygenases and cyclooxygenases are key mediators of aracidonic acid metabolism, and one of each, 12-LOX and COX2, are upregulated by Mirk. Thus Mirk kinase was found expressed in mature fast twitch skeletal muscle, where it could reduce ROS levels arising from contraction by upregulating the expression of a series of antioxidant genes.

**Table I:** qRT-PCR Determinations of Oxidative Stress Genes Downregulated When Mirk Depleted in SU86.86 pancreatic cancer cells

Oxidative Stress Genes	Average ΔC_t_		2 ^ -Δ Ct		Fold Expression
	+DOX	−DOX	+DOX	−DOX	+DOX/− DOX
glutathione peroxidase2 (GPX2)	8.68	7.06	2.4E-03	7.5E-03	−3.07
peroxidasin (PXDNL)	12.48	10.16	1.8E-04	8.7E-04	−4.99
sulfiredoxin (SRXN1)	6.08	5.06	1.5E-02	3.0E-02	−2.03
peroxiredoxin 2 (PRDX2)	1.68	0.96	3.1E-01	5.1E-01	−1.65
COX2	4.78	4.06	3.6E-02	6.0E-02	−1.65
arachidonate 12- lipoxygenase (12-LOX)	10.08	9.36	9.2E-04	1.5E-03	−1.65
superoxide dismutase 2 (SOD2)	1.08	0.56	4.7E-01	6.8E-01	−1.43*
superoxide dismutase 3 (SOD3)	4.68	4.16	3.9E-02	5.6E-02	−1.43*

* genes confirmed by northern analysis to be downregulated when Mirk depleted (18)

**Table:** Five Housekeeping genes (HKG) used as controls

Genes	Average ΔC_t_		2 ^ -Δ Ct		Fold Expression
	+DOX	-DOX	+DOX	-DOX	+DOX/− DOX
beta 2-microglobulin	−0.92	−1.14	1.9E+00	2.2E+00	−1.16
hypoxanthine phospho- ribosyltransferase 1	4.78	5.06	3.6E-02	3.0E-02	1.21
ribosomal protein L13a	0.68	0.36	6.2E-01	7.8E-01	−1.25
GAPDH	−2.02	−2.04	4.1E+00	4.1E+00	−1.01
beta actin	−2.52	−2.24	5.7E+00	4.7E+00	1.21

The formula used to calculate the relative gene expression level (2 ^ -Δ Ct):

Δ Ct = C_t_ (GOI) – avg. (C_t_ (HKG)), where GOI is each gene of interest, and HKG are the housekeeping genes.

## DISCUSSION

The Mirk kinase gene has been localized to the 19q13 amplicon [12] is amplified in a subset of pancreatic cancers and ovarian cancers, and less frequently in colon cancers, and is upregulated in the majority of these cancers [19], [20]. Mirk is expressed at very low levels in most normal diploid cells except for skeletal muscle. The muscle cell function of Mirk kinase selected for by cancer cells was unknown, although Mirk knockdown reduced myoblast viability by 75% [11]. In the current study we show that Mirk kinase depletion and Mirk kinase inhibition increase the amount of toxic ROS induced in differentiating C2C12 myoblasts and in postmitotic cultures of fused, contractile myotubes that model skeletal muscle. The results of this study showed that Mirk is localized in fast twitch skeletal muscles. Such muscle endogenously produces ROS in response to repeated contractions. Thus Mirk combats the ROS generated by contraction in skeletal muscle, so some cancer cells upregulate Mirk or amplify the Mirk gene to combat ROS.

ROS are oxygen containing chemical species with reactive chemical properties, such as hydroxyl radicals, which contain an unpaired electron and the free radical superoxide. Hydrogen peroxide is produced in contracting muscle, breaking down to ROS species, which can have diverse effects on myoblasts, such as inducing mitochondrial fragmentation [21]. Many investigators have reported that ROS levels increase during myoblast differentiation. Mitochondria also are a source of ROS in skeletal muscle cells [22], [23]. ROS generation within single intact muscle fibers was cytosolic, with a role for NADPH oxidase-derived ROS during contractile activity [24]. Cancer cells often exhibit higher levels of ROS than normal cells because of increased metabolism and oncogenic stimulation, so are under increased oxidative stress. Genes which detoxify superoxide (superoxide dismutases 2 and 3) and which prevent the generation of hydroxyl radical (ferroxidase/ceruloplasmin) were found to be upregulated in SU86.86 pancreatic cancer cells in prior work [18] and in this study (Table 1), and in each of four ovarian cancer cell lines [25] through Mirk. These genes work together to reduce ROS. Superoxide dismutases detoxify superoxide resulting in hydrogen peroxide, which in turn can either be metabolized to water or to hydroxyl radical through the Fenton reaction if Fe++ is available. Conversion to hydroxyl radical is blocked by ferroxidase that converts Fe++ to Fe+++. Mirk is a co-activator for several transcription factors and increases the expression of these antioxidant genes and 6 others in pancreatic cancer cells [18], Table I. Recently peroxiredoxin-2 upregulated by NFkappaB was shown to attenuate oxidative stress during the differentiation of C2C12 cells [26]. Interestingly, peroxiredoxin-2 expression was decreased when Mirk was depleted (Table I), suggesting the Mirk may play a role in control of expression of this antioxidant gene during myogenesis. Depletion of Mirk in C2C12 cells, followed by sorting of the transfected cells, showed that pyruvate dehydrogenase kinase 4 (PDK4) mRNA was upregulated 6 fold when Mirk was depleted [10]. PDK4 inhibits one component of the pyruvate dehydrogenase complex that transforms pyruvate into acetyl-CoA. This function of the PD complex results in the generation of NADH, which reduces ROS, so increasing PDK4 levels will inhibit NADH generation and lead to increased ROS levels. Mirk kinase inhibition also led to increased Pdk4 protein (data not shown). Thus these Mirk-upregulated genes working together increase antioxidant potential while minimizing hydroxyl production.

## MATERIALS AND METHODS

### Materials

Rabbit polyclonal antibodies were raised to unique sequences at the N-terminus and C-terminus of Mirk and affinity purified [12]. The C-terminal antibody was labeled with Alexa Fluor 594 using the Xenon Rabbit IgG Labeling Kit (Molecular Probes) for use in direct immunofluorescence experiments. Alexa Fluor 488 and 594 (highly cross-adsorbed) secondary antibody conjugates and Alexa Fluor 594 phalloidin were purchased from Molecular Probes. Mirk kinase inhibitors EHT5372, EHT6840 and EHT411 were the gifts of Diaxonhit, Paris, France. Antibodies to myogenin, and actin were purchased from Santa Cruz Biotechnology. Tissue culture reagents were obtained from Mediatech. Other reagents were obtained from Sigma.

### Cell Culture

C2C12 mouse skeletal myoblasts were obtained from the ATCC and were maintained in growth medium (Dulbecco's modified Eagle's medium, 4 mM L-glutamine, 4.5 g/L glucose, containing 20% fetal bovine serum) and induced to undergo differentiation by switching to differentiation medium (; Dulbecco's modified Eagle's medium containing 2% horse serum). Cells were used for experiments only from passages 3-10 from our frozen stocks.

### Immunofluorescence of Human Muscle Frozen Sections

Anonymous samples of flash-frozen human muscle tissue on slides were obtained from the Department of Pathology, SUNY Upstate Medical University in accordance with institutional review procedures for clinical specimen use. Slides were thawed at room temperature for 15 minutes, hydrated with PBS for 15 minutes, and then fixed in 4% paraformaldehyde for 1 minute at room temperature, or ice-cold acetone for 10 minutes at 0°C. Slides were rinsed 3 x 1 minute with PBS. The sections were then demarcated with a PAP pen (Zymed) and permeabilized for 20 min with 0.2% Triton X-100 in PBS (wash buffer; used in all subsequent steps), and blocked with 10% normal goat serum/0.2% Triton/PBS for 30 minutes. Mirk was visualized with polyclonal antibody to either the N- or C-terminus of Mirk (both at 1:500 dilution for 1 hour). Non-specific rabbit IgG diluted to an equivalent mass/concentration was used as a negative control. All labelling incubations were diluted in 10% normal goat serum/0.2% Triton/PBS. After 3 x 5 minute washes, cells were incubated for 30 minutes with a 1:1000 dilution of goat anti-rabbit IgG antibody Alexa Fluor conjugate. Sections were washed 2 x 5 minutes. In some experiments, sections were incubated for 20 minutes with 2 units of Alexa Fluor 594 phalloidin diluted in 3%BSA/PBS, and washed twice. Nuclei were counterstained with a 5 minute incubation with a 2 ng/ml solution of 4', 6'-diamidino-2-phenylindole hydrochloride (DAPI). Following 3 x 5 minute washes, slides were rinsed with distilled water, blotted dry and mounted with Biomedia Gel/Mount. Images were obtained as described below.

### Fiber Type Distribution of Mirk Kinase

Formalin-fixed paraffin-embedded sections (3μm) were deparaffinized, hydrated and antigen retrieval was performed by autoclaving 20 minutes in 10 mM citrate pH 6.0. The tissue was permeabilized with 0.2% Triton X-100 in PBS (PBST) for 20 minutes and non-specific interactions were controlled by a 30 minute incubation with 10% normal goat serum in PBST. Slides were incubated for 1 hour at room temperature with polyclonal anti-Mirk C-terminal antibody (1:500 dilution) combined with either monoclonal rabbit antibody against either skeletal slow-twitch myosin (1:1000, Sigma M 8421) or fast-twitch myosin (1:250, Sigma 4276). Non-specific mouse and rabbit IgG diluted to an equivalent mass/concentration was used as a negative control. All labelling incubations were diluted in 10% normal goat serum/0.2% Triton/PBS. After 3 x 5 minute washes, cells were incubated for 1 hour with 1:500 dilutions of goat anti-rabbit Alexa Fluor 488 conjugate and goat anti-mouse Alexa Fluor 594. Monochrome fluorescence images were obtained at 200X using a Diagnostic Instruments SPOT RT camera mounted on a Nikon Eclipse E50i fluorescent microscope. SPOT RT Software v4.09 was used to pseudocolor the images, adjust the RGB histogram and merge the images. Image manipulation consisted of resetting the zero point of the RGB histogram of the green and red fluorescent channels to stretch the darker areas of the image yielding a uniform black background consistent with the image viewed through the microscope.

Subcellular fractionation, Mirk immune complex kinase reaction, western blotting, determination of ROS, and Mirk depletion by RNA interference were performed as described previously [10], [13], [18].

### qRT-PCR

SU.86.86 pancreatic cell pools containing deoxycycline-inducible lentiviral shMirk constructs were depleted of Mirk (18) and total mRNA populations of antioxidant stress gene transcripts (PAHS-065) were compared by qRT-PCR data array analysis as described, and normalized to the expression levels of 5 housekeeping genes (SABiosciences).
